# Prognostic Value of Apparent Diffusion Coefficient in Oropharyngeal Carcinoma

**DOI:** 10.1007/s00062-021-01014-4

**Published:** 2021-04-20

**Authors:** Heli J. Sistonen, Katri Aro, Timo Atula, Lauri Jouhi, Riikka Lindén, Laura Tapiovaara, Venla Loimu, Antti Markkola

**Affiliations:** 1 Radiology, HUS Diagnostic Center, University of Helsinki and Helsinki University Hospital, Haartmaninkatu 4, 00029 HUS Helsinki, Finland; 2 Department of Otorhinolaryngology—Head and Neck Surgery, University of Helsinki and Helsinki University Hospital, Kasarmikatu 11–13, 00029 HUS Helsinki, Finland; 3 Department of Oncology, University of Helsinki and Helsinki University Hospital, Paciuksenkatu 3, 00029 HUS Helsinki, Finland

**Keywords:** Human papilloma virus, Magnetic resonance imaging, Diffusion weighted imaging, Radiotherapy, Tumor volume

## Abstract

**Purpose:**

To investigate clinical and radiological factors predicting worse outcome after (chemo)radiotherapy ([C]RT) in oropharyngeal squamous cell carcinoma (OPSCC) with a focus on apparent diffusion coefficient (ADC).

**Methods:**

This retrospective study included 67 OPSCC patients, treated with (C)RT with curative intent and diagnosed during 2013–2017. Human papilloma virus (HPV) association was detected with p16 immunohistochemistry. Of all 67 tumors, 55 were p16 positive, 9 were p16 negative, and in 3 the p16 status was unknown. Median follow-up time was 38 months. We analyzed pretreatment magnetic resonance imaging (MRI) for factors predicting disease-free survival (DFS) and locoregional recurrence (LRR), including primary tumor volume and the largest metastasis. Crude and p16-adjusted hazard ratios were analyzed using Cox proportional hazards model. Interobserver agreement was evaluated.

**Results:**

Disease recurred in 13 (19.4%) patients. High ADC predicted poor DFS, but not when the analysis was adjusted for p16. A break in RT (hazard ratio, HR = 3.972, 95% confidence interval, CI 1.445–10.917, *p* = 0.007) and larger metastasis volume (HR = 1.041, 95% CI 1.007–1.077, *p* = 0.019) were associated with worse DFS. A primary tumor larger than 7 cm^3^ was associated with increased LRR rate (HR = 4.861, 1.042–22.667, *p* = 0.044). Among p16-positive tumors, mean ADC was lower in grade 3 tumors compared to lower grade tumors (0.736 vs. 0.883; *p* = 0.003).

**Conclusion:**

Low tumor ADC seems to be related to p16 positivity and therefore should not be used independently to evaluate disease prognosis or to choose patients for treatment deintensification.

## Introduction

Oropharyngeal squamous cell carcinoma (OPSCC) subdivides into human papilloma virus (HPV)-associated and non-HPV-associated cancer groups. Immunohistochemical (IHC) overexpression of p16 is a surrogate marker for HPV and used in the current TNM classification [1] and the World Health Organization (WHO) classification of head and neck tumors [2]. A HPV-associated OPSCC usually responds well to (chemo)radiation ([C]RT), and treatment deintensification trials are underway to reduce treatment adverse effects [3, 4]. In a small subset of patients, however, the disease recurs making salvage surgery more demanding.

Prior to treatment, patients undergo multiplanar radiological imaging. Some radiological features show prognostic impact, such as volume of the primary tumor and metastasis, lymph node cystic or matted morphology, and extranodal extension (ENE) [5–8]. Many reports link primary tumor relatively high apparent diffusion coefficient (ADC) in magnetic resonance imaging (MRI) with lower radiosensitivity [7, 9–16], although some conflicting evidence has emerged [17–19]. These studies include cancers from multiple head and neck sites, however, and all but one overlook HPV association [17]. This confounds results because HPV positive tumors tend to have lower ADC [20–24], and respond better to (C)RT.

Our aim was to investigate the prognostic effect of clinical and radiological variables, including ADC, in pretreatment MRI in an OPSCC population treated with (C)RT with curative intent. These features were then compared with the tumor p16 status. We hypothesized that ADC would serve to estimate treatment response and prognosis after (C)RT and improve management guidelines. This might support the decision to exclude patients who seem to have a worse prognosis from the deintensification protocols, and lead to offering them more extensive treatment.

## Material and Methods

### Study Design and Patient Selection

**Fig. 1:**
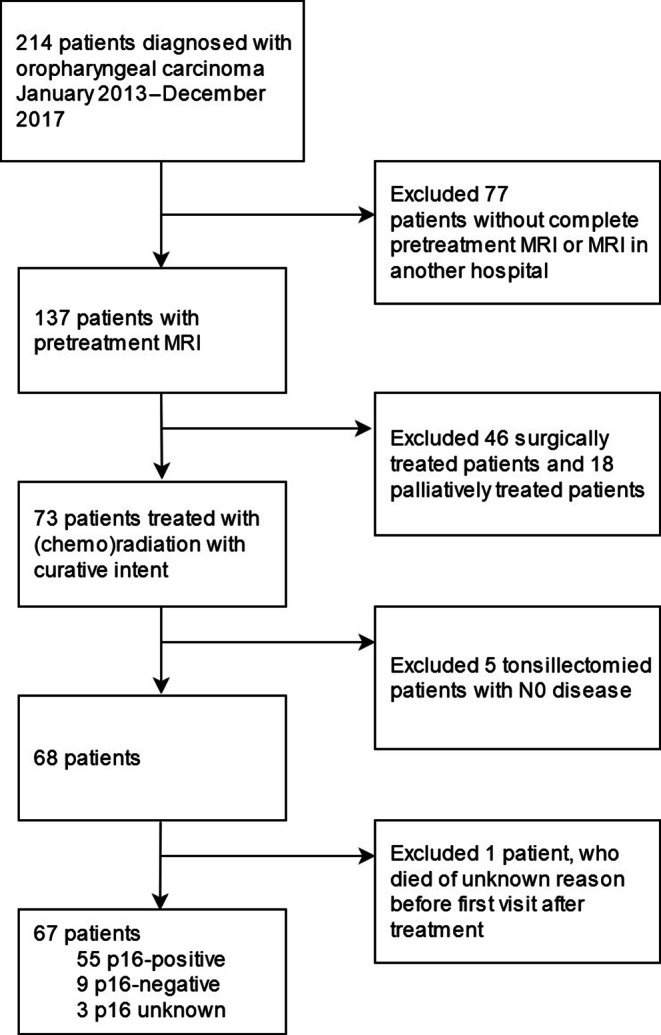
Patient exclusion chart

We included OPSCC patients treated at the Helsinki University Hospital Head and Neck Center, diagnosed between January 2013 and December 2017. Our multidisciplinary tumor board reviewed the diagnostics, staging, and treatment plan for all patients. The follow-up data were collected in December 2018. Inclusion criteria were histologically proven OPSCC, available pretreatment diagnostic MRI, and (C)RT with curative intent. We excluded patients with previous head and neck cancer (HNC) or distant metastasis at presentation (Fig. 1). The final study cohort consisted of 67 patients, of whom 55 had p16 positive disease, 9 had p16 negative disease, and in 3 patients the result of p16 status was unavailable. We chose the years from 2013 onwards, when the diffusion-weighted imaging (DWI) sequence was added to our institute’s tumor imaging protocol. The protocol remained mainly constant during the study period and DWI was available for 63 patients (for 52 of the 55 p16 positive patients).

### Treatment and Follow-up

In a small subset of patients, the tumor was biopsied via tonsillectomy after MRI. We included 8 of these patients who had undergone a primary tonsillectomy with metastatic lymph nodes (N + disease), as these patients received the same locoregional treatment as others and they are a typical patient population receiving definitive (C)RT. Patients who underwent tonsillectomy and had no neck metastases (N0 disease) were excluded as they had no macroscopic tumor left prior to the RT. All patients were treated with intensity-modulated RT (IMRT), range 56–70 Gy; 66 patients received the treatment conventionally fractionated with 2 Gy daily fractions and 1 with simultaneous integrated boost (SIB). RT was delivered to the primary tumor and cervical lymph node areas. Chemotherapy was administered with weekly cisplatin 40 mg/m^2^ (maximum of 6 doses).

After treatment, patients underwent regular follow-up appointments and positron emission tomography with computed tomography (PET-CT) at 3 months. Additional diagnostic and therapeutic procedures were planned in cases of suspicion of a residual or recurrent disease. Locoregional recurrence (LRR) was defined either as a residual disease (a persistent disease in follow-up PET-CT and first clinical examination with no remission in between) or a later recurrent disease (a reappearance of the tumor at the primary site or metastases after a disease-free period). Biopsy or clear imaging evidence with progression confirmed the recurrence. Residual disease was found in 7 (10.4%) and later recurrent disease in 6 (9.0%). Median time to recurrence was 98 days. Follow-up time was calculated from the last day of treatment to the last follow-up visit or death, whichever occurred first. Salvage surgery included primary tumor area, or neck dissection, or both depending on the site of the recurrence.

### Pretreatment MRI and Analysis

Our standard tumor imaging protocol consisted of a localizer sequence, axial turbo spin echo (TSE) T2, axial fat saturated T2, axial TSE T1, axial and coronal fat saturated T1 with gadolinium, and axial echo-planar DWI with b‑values of 0, 500, and 1000 s/mm^2^. ADC maps were generated from DWI sequences. The patients were imaged with six different MRI scanners: two were 1.5 T Siemens Avanto fit (Siemens Healthcare, Erlangen, Germany), two were 1.5 T Siemens Avanto (Siemens Healthcare), one was 1.5 T GE Signa HDxt (GE Healthcare, Chicago, IL, USA), and one was 3 T Siemens Verio (Siemens Healthcare). Dedicated head and neck coils were applied. Two experienced head and neck radiologists (H. S. and R. L.) analyzed the images, blinded to tumor p16 status and treatment outcome.

### Clinical and Radiological Variables

All patient data were gathered from hospital records (Tables 1 and 2). Tumors primarily staged by the 7th edition of the UICC system were retrospectively restaged according to the UICC 8th edition [1]. The p16 IHC served for determining the tumor HPV association. The criteria of the size of the metastatic node were minimum axial diameter of 10 mm and 11 mm for the digastric node [25]. Heterogeneous nodes were counted as metastatic. Cystic metastases were defined to have a thin (< 2 mm) enhancing capsule and internal homogeneous fluid content [26], or as an intranodal cystic space with more than 70% of the border smoothly delineated [27]. Otherwise, a fluid-containing metastatic node was defined as necrotic. Lymph node location was documented according to the American Academy of Otolaryngology–Head and Neck Surgery (AAO–HNS) 2002 classification [28].

**Table 1:** Patient demographics, treatment data and treatment results, stratified by p16

	p16+ *n* = 55	p16− *n* = 9	All *n* = 67	P‑value (p16+ vs. p16−)
Age, years, mean (range)	60.8 (41–79)	66.7 (54–77)	62 (41–79)	0.113
*Gender, %*				
Female	9 (16.4)	7 (77.8)	14 (20.9)	0.646
Male	46 (83.6)	2 (22.2)	53 (79.1)	
*Smoking, %*				
No	21 (38.2)	1 (11.1)	22 (32.8)	**0.001**
Yes	13 (23.6)	8 (88.9)	24 (35.8)	
Before	20 (36.4)	–	20 (29.9)	
Data missing	1 (1.8)	–	1 (1.5)	
*Alcohol use, %*				
No	51 (92.7)	5 (55.6)	58 (86.6)	**0.012**
Yes	2 (3.6)	3 (33.3)	5 (7.5)	
Before	2 (3.6)	1 (11.1)	4 (6.0)	
*Tumor site, %*				
Tonsils	36 (65.5)	7 (77.8)	43 (64.2)	0.154
Base of tongue	18 (32.7)	1 (11.1)	21 (31.3)	
Posterior wall of the oropharynx	1 (1.8)	1 (11.1)	3 (4.5)	
*Grade, %*				
1	–	–	1 (1.5)	**<** **0.001**
2	7 (12.7)	7 (77.8)	15 (22.4)	
3	45 (81.8)	2 (22.2)	48 (71.6)	
Data missing	3 (5.5)	–	3 (4.5)	
*T‑stage (UICC8), %*				
T1	12 (21.8)	–	–	**0.003**
T2	27 (49.1)	2 (22.2)		
T3	6 (10.9)	1 (11.1)		
T4	10 (18.2)	–		
T4a	–	5 (55.6)		
T4b	–	1 (11.1)		
*N‑stage (UICC8), %*				
N0	6 (10.9)	3 (33.3)	–	0.055
N1	43 (78.2)	–		
N2	6 (10.9)	–		
N2b	–	1 (11.1)		
N2c	–	2 (22.2)		
N3b	–	3 (33.3)		
*Stage, %*				
I	38 (69.1)	–	–	**<** **0.001**
II	7 (12.7)	2 (22.2)		
III	9 (16.4)	1 (11.1)		
IV	1 (1.8)	–		
IVa	–	3 (33.3)		
IVb	–	3 (33.3)		
*Treatment modality, %*				
Chemoradiation	50 (90.9)	8 (88.9)	61 (91.0)	1.000
Radiation	5 (9.1)	1 (11.1)	6 (9.0)	
*Time interval between diagnostic MRI and start of treatment, days, mean, range*	43 (19–75)	45 (29–91)	44 (19–91)	0.575
*Pause in radiation treatment, %*				
No	44 (80.0)	7 (77.8)	54 (80.6)	1.000
Yes	11 (20.0)	2 (22.2)	13 (19.4)	
*Chemotherapy finished as planned, %*	*n* = 50	*n* = 8	*n* = 61	0.124
Yes	34 (68.0)	3 (37.5)	39 (63.9)	
No	16 (32.0)	5 (62.5)	22 (36.1)	
*Follow-up, months*				
Mean	36	22	34	**0.046**
Median	38	18	38	
Range	3–61	2–54	2–61	
*Disease recurrence, %*	7 (12.7)	6 (66.7)	13 (19.4)	**0.001**
Residual disease at 3 months	3 (5.5)	4 (44.4)	7 (10.4)	
Later recurrence	4 (7.2)	2 (22.2)	6 (9.0)	
*Time to recurrence, days, median, range*	216 (71–885)	85 (64–412)	98 (64–885)	0.138
*Locoregional recurrence, %*	5 (9.1)	6 (66.7)	11 (16.4)	**<** **0.001**
Local	1 (1.8)	5 (55.6)	6 (9.0)	
Neck	4 (7.3)	3 (33.3)	6 (9.0)	
*Distant metastasis, %*	4 (7.3)	2 (22.2)	6 (9.0)	0.196
*Overall survival, %*	49 (89.1)	3 (33.3)	54 (80.6)	**<** **0.001**
*Disease-free survival, %*	46 (83.6)	2 (22.2)	50 (74.6)	**<** **0.001**
*Disease-specific survival, %*	51 (92.7)	4 (44.4)	58 (86.6)	**0.002**

**Table 2:** Radiological variables, stratified by p16, and interobserver correlations

Variables	p16+ *n* = 55	p16− *n* = 9	All *n* = 67	*p*-value (difference between p16+ and p16−)	Interobserver correlation
**Primary tumor**					
Volume, cm^3^, mean (range)	8.1 (0.7–35.3)	18.6 (3.6–39.8)	10.7 (0.7–92.5)	0.034	–
ADC_mean_, 10^−3^ mm^2^/s (range)	0.747 (0.496–1.233)	0.966 (0.838–1.045)	0.784 (0.496–1.233)	< 0.001	0.783
ADC_min_, 10^−3^ mm^2^/s (range)	0.595 (0.424–1.005)	0.761 (0.606–0.873)	0.621 (0.400–1.005)	< 0.001	0.797
Necrosis, %	6 (10.9)	3 (33.3)	10 (14.9)	0.106	0.203
Ulceration, %	23 (41.8)	7 (77.8)	32 (47.8)	0.071	0.302
Inflammation, %	36 (65.5)	7 (77.8)	45 (67.1)	0.706	0.451
Exophytic growth, %	34 (61.8)	2 (22.2)	37 (55.2)	0.035	0.528
Well-delineated border, %	36 (65.5)	3 (33.3)	39 (58.2)	0.137	0.620
Intense enhancement, %	25 (45.5)	6 (66.7)	32 (47.8)	0.296	0.397
Muscle invasion, %	20 (36.4)	6 (66.7)	28 (41.8)	0.142	0.624
**Neck metastasis**					
*Number of metastatic nodes*					
Mean	2.6	2.9	2.6	0.732	0.517
Median	2	3	2		
Range	0–10	0–6	0–10		
*Location, %*					
Ipsilateral	42 (76.4)	3 (33.3)	46 (68.7)	0.179	0.950
Contralateral	1 (1.8)	–	1 (1.5)		
Bilateral	8 (14.5)	3 (33.3)	12 (17.9)		
*Diameter, cm*					
Mean	3.6	2.8	3.6	0.092	–
Range	1.2–11.1	2.2–4.9	1.2–11.1		
*Volume, cm*^*3*^	15.2 (0.6–56.0)	14.4 (2.8–37.8)	14.9 (0.6–56.0)	0.747	–
*ADC*_*mean*_*, 10*^*−3*^ *mm*^*2*^*/s (range)*	0.783 (0.537–1.483)	0.955 (0.818–1.141)	0.802 (0.537–1.483)	0.013	0.913
*ADC*_*min*_*, 10*^*−3*^ *mm*^*2*^*/s (range)*	0.622 (0.366–0.974)	0.797 (0.628–0.904)	0.638 (0.366–0.974)	0.007	0.820
*Extranodal extension, %*	35 (63.6)	5 (55.6)	41 (61.2)	0.718	0.708
*Matted nodes, %*	18 (32.7)	3 (33.3)	21 (31.3)	1.000	0.895
*Solid nodes, %*	9 (16.4)	9 (100)	9 (13.4)	0.337	0.872
*Necrotic nodes, %*	21 (38.2)	3 (33.3)	24 (35.8)	1.000	0.499
*Cystic nodes, %*	19 (34.5)	3 (33.3)	23 (34.3)	1.000	0.516

*ADC* apparent diffusion coefficient

The volume of the primary tumor and the largest single metastasis or matted metastatic lymph node mass were measured by manually drawing the region of interest (ROI) in all MRI slides containing the tumor. Volume was measured primarily from the ADC maps, which usually well differentiate the tumor and surrounding edema. The T2 and T1 gadolinium-enhanced fat saturated images were used as a reference. If ADC maps were unavailable or tumor was not well delineated, T1 gadolinium-enhanced fat saturated images were used instead. Prior to volume measurement, MR images were exported from our picture archiving and communication system (Agfa Impax 6.7, Agfa Healthcare, Mortsel, Belgium) and anonymized. Volume measurement was performed with third party 3D software (3D Slicer version 4.10.1, www.slicer.org).

### DWI Analysis

**Fig. 2:**
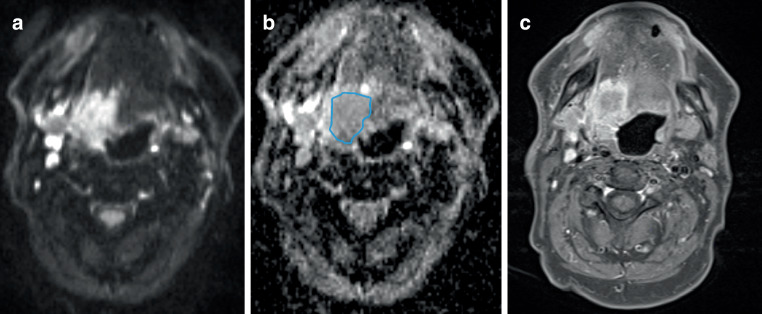
Measuring apparent diffusion coefficient (ADC) region of interest (ROI). **a** Axial diffusion weighted imaging, **b** corresponding ADC map and **c** axial T1 fat saturated gadolinium-enhanced images show a tonsillar tumor infiltrating to the tongue on the right. The ROI was drawn on the ADC map along the tumor borders, excluding the surrounding inflammation and necrotic areas, on the most central slice of the tumor. The ADC was 1.097 × 10^−3^ mm^2^/s

The ADC_mean_ of the primary tumor and metastatic lymph node was measured by manually drawing an ROI on the single slice most central to the tumor (Fig. 2). The T2 and T1 fat saturated gadolinium-enhanced images served as a reference to exclude necrotic areas. ADC_min_ was measured with the smallest available 0.24 cm^2^ ROI from the most hypointense part of the tumor.

The ADC values differ between MRI systems and ADC value of the cervical spinal cord may be used to compare between these differences [29]. In an attempt to estimate the ADC values across different scanners, we measured ADC at the level of cervical spinal cord in 3 central slices to calculate a mean value (ADC_myelum_) for each examination.

### Statistical Analysis

The main clinical outcome measures were disease-free survival (DFS) and LRR rate, as we found them to be most representative of the treatment effect in our study setting. Time to event or censoring was counted from the end date of (C)RT. We first conducted univariable Cox proportional hazards model to find potential prognostic factors, then adjusted the results with the tumor p16-status.

Differences in variable values between p16-positive and p16-negative tumors were compared with χ^2^-testor Fisher’s exact test with categorical variables and Mann-Whitney U‑nonparametric test with ordinal or continuous variables, which in our study were all non-normally distributed. Interobserver agreement for categorical radiological variables was calculated with Cohen’s kappa and in continuous variables with intraclass correlation coefficient (ICC). Values under 0.4 indicated poor agreement, values between 0.4 and 0.75 indicated moderate agreement, and values 0.75 and over indicated excellent agreement [30]. Variables with poor interobserver agreement were not studied.

The differences between MRI systems were evaluated by comparing the mean value of ADC_myelum_ for each MRI system examinations with Kruskal-Wallis nonparametric test. A *p*-value of < 0.05 was considered significant.

The data analysis tool was SPSS Statistics 25 software (SPSS Inc., Chicago, IL, USA). Survival curves were drawn using GraphPad Prism (version 9.0 for Windows, Graphpad software, La Jolla, CA, USA, www.graphpad.com).

## Results

### Patients

Table 1 presents patient demographics, treatment outcome, and survival data, stratified by tumor p16 status. In patients with p16-negative disease, both overall survival (OS) and DFS were noticeably inferior. Of the 13 patients with LRR, further treatment included surgery with curative intent in 5 patients, palliative radiotherapy in 1 patient, palliative chemotherapy in 2 patients, palliative CRT in 2 patients, and palliative symptomatic treatment in 2 patients. One patient died of hemorrhage at the time of diagnosis of the recurrent tumor.

Thirteen patients experienced a treatment break in RT ranging from 2–18 days (median 6 days). In total, 39 patients received chemotherapy as planned. Three patients received a chemotherapy agent other than cisplatin.

### Prognostic Value of Clinical and Radiological Variables for DFS (Table 3)

In univariable analysis, patients with p16-negative tumor had a 7.7-fold risk for disease recurrence or death compared with patients with p16-positive tumor. Smoking at diagnosis and number of pack years were also significantly associated with worse DFS. Interruptions in RT and incomplete chemotherapy were significantly associated with worse DFS. After adjusting for these variables with p16, only interruption of RT remained significant in DFS analysis.

**Table 3:** Crude and p16-adjusted hazard ratios (HR) for disease-free survival in Cox proportional hazards regression model

Variable	All patients *n* = 67 Crude HR (95% CI)	*p*	p16-adjusted HR (95% CI)	*p*
Age	1.040 (0.986–1.096)	0.147	1.044 (0.984–1.107)	0.155
Smoking (at diagnosis vs. no/earlier)	3.178 (1.152–8.765)	**0.026**	1.122 (0.236–5.333)	0.885
Pack years	1.039 (1.005–1.074)	**0.025**	1.025 (0.981–1.071)	0.279
Alcohol use (current vs. previous/never)	2.771 (0.794–9.664)	0.110	1.249 (0.294–5.299)	0.763
T‑stage (7th ed. T3–4 vs. T1–2)	2.140 (0.825–5.553)	0.118	1.297 (0.448–3.751)	0.632
T‑stage (8th ed. T3–4 vs. T1–2)	1.731 (0.668–4.490)	0.259	0.980 (0.334–2.875)	0.971
N‑stage (N + vs. N0)	0.305 (0.112–0.828)	**0.020**	0.585 (0.180–1.898)	0.372
N‑stage (N2+ vs. N0–1)	2.026 (0.712–5.760)	0.186	0.921 (0.259–3.284)	0.900
Stage (III–IV vs. I–II)	2.050 (0.742–5.663)	0.166	0.950 (0.295–3.063)	0.932
p16 (negative vs. positive)	7.674 (2.832–20.800)	**<** **0.001**	–	–
Grade (grade 3 vs. grades 1–2)	0.268 (0.101–0.716)	**0.009**	0.799 (0.203–3.149)	0.749
Break in radiation treatment (yes vs. no)	3.583 (1.358–9.457)	**0.010**	3.972 (q)	**0.007**
Incomplete chemotherapy (yes vs. no)	3.086 (1.096–8.693)	**0.033**	1.936 (0.638–5.877)	0.243
*Radiological variables*				
Primary tumor volume	1.004 (0.976–1.033)	0.783	1.009 (0.966–1.054)	0.690
Primary tumor volume (> 7 cm^3^ vs. ≤ 7 cm^3^)	2.209 (0.816–5.975)	0.119	2.131 (0.762–5.959)	0.149
Primary tumor transverse diameter	1.399 (0.952–2.054)	0.087	1,618 (0.936–2.796)	0.085
Primary tumor ADC_mean_	21.780 (1.151–412.019)	**0.040**	0.751 (0.007–83.525)	0.905
Primary tumor ADC_min_	40.775 (1.647–1009.699)	**0.024**	0.933 (0.008–107.248)	0.977
Muscle invasion (yes vs. no)	2.785 (1.043–7.439)	**0.041**	2.342 (0.818–6.701)	0.113
Invasion depth	1.504 (1.006–2.247)	**0.047**	1.329 (0.874–2.022)	0.184
Metastasis volume	1.036 (1.003–1.069)	**0.034**	1.041 (1.007–1.077)	**0.019**
Metastasis volume (> 13 cm^3^ vs. ≤ 13 cm^3^)	2.540 (0.825–7.820)	0.104	2.384 (0.758–7.492)	0.137
Metastasis ENE	0.659 (0.254–1.710)	0.391	0.827 (0.303–2.259)	0.712
Metastasis ADC_mean_	2.128 (0.096–47.281)	0.633	0.990 (0.020–50.115)	0.996
Metastasis ADC_min_	6.658 (0.079–563.032)	0.402	0.974 (0.005–202.478)	0.992

*ADC* apparent diffusion coefficient, *ENE* extranodal extension, *CI* confidence interval

**Fig. 3:**
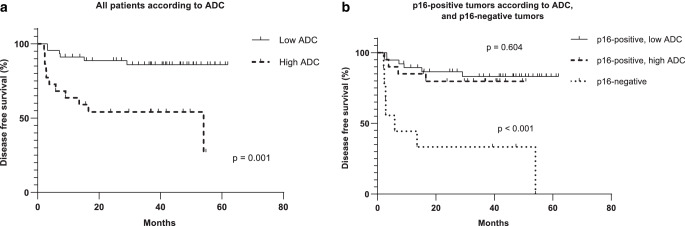
**a** Survival curves for all patients (p16-positive and p16-negative tumors). Disease-free survival (DFS) in two groups of primary tumor apparent diffusion coefficient (ADC_mean_) at or higher than 0.836 × 10^−3^ mm^2^/s, and lower than 0.836 × 10^−3^ mm^2^/s, *p* = 0.001. **b** Survival curves for patients with p16-positive and p16-negative tumors. In p16-positive patients, disease-free survival (DFS) was represented in two groups of primary tumor apparent diffusion coefficient (ADC_mean_): at or higher than 0.772 × 10^−3^ mm^2^/s, and lower than 0.772 × 10^−3^ mm^2^/s, *p* = 0.604. Difference between mean ADC_mean_ between p16-positive and p16-negative tumors was statistically significant (*p* < 0.001)

In univariable analysis, metastasis volume, muscle invasion, and depth of muscle invasion indicated significantly worse DFS. Primary tumor higher ADC_mean_ and ADC_min_ were associated with worse DFS. After adjusting these variables with p16, only metastasis volume remained significant in DFS analysis. Larger metastasis volume but not primary tumor volume, was associated with the occurrence of distant metastasis, regardless of p16 status. The HR was 1.059 (95% CI 1.011–1.110, *p* = 0.015). Survival curves stratified by ADC are presented in Fig. 3a, b.

### Prognostic Value of Clinical and Radiological Variables in LRR (Table 4)

In univariable analysis, LRR rate was associated with p16 and higher T‑stage, stage, and grade. After adjusting these variables with p16, none of these clinical variables remained significant in LRR rate analysis.

**Table 4:** Crude and p16-adjusted hazard ratios (HR) for locoregional recurrence rate in Cox proportional hazards regression model

Variable	All patients *n* = 67 Crude HR (95% CI)	*p*	p16-adjusted HR (95% CI)	*p*
Age	1.055 (0.986–1.128)	0.119	1.047 (0.974–1.125)	0.212
Smoking (at diagnosis vs. no/earlier)	2.270 (0.692–7.445)	0.176	0.194 (0.11–3.396)	0.261
Pack years	1.044 (0.998–1.092)	0.060	1.027 (0.967–1.089)	0.387
Alcohol use (current vs. previous/never)	2.912 (0.629–13.488)	0.172	1.524 (0.291–7.973)	0.618
T‑stage (7th ed. T3–4 vs. T1–2)	4.983 (1.320–18.807)	**0.018**	2.975 (0.715–12.379)	0.134
T‑stage (8th ed. T3–4 vs. T1–2)	3.388 (0.991–11.584)	0.052	1.861 (0.484–7.150)	0.366
N‑stage (N + vs. N0)	0.447 (0.118–1.688)	0.235	10.462 (2.962–36.959)	0.735
N‑stage (N2+ vs. N0–1)	2.678 (0.784–9.155)	0.116	0.771 (0.182–3.259)	0.724
Stage (III–IV vs. I–II)	3.912 (1.192–12.834)	**0.024**	1.550 (0.382–6.285)	0.540
p16 (negative vs. positive)	11.170 (3.378–36.931)	**<** **0.001**	–	–
Grade (grade 3 vs. grades 1–2)	0.231 (0.071–0.759)	**0.016**	0.648 (0.132–3.186)	0.593
Break in radiation treatment (yes vs. no)	2.639 (0.772–9.024)	0.122	2.918 (0.487–17.474)	0.241
Incomplete chemotherapy (yes vs. no)	1.905 (0.551–6.583)	0.308	1.141 (0.308–4.225)	0.843
*Radiological variables*				
Primary tumor volume	1.016 (0.989–1.045)	0.242	1.023 (0.977–1.072)	0.328
Primary tumor volume (> 7 cm^3^ vs. ≤ 7 cm^3^)	5.282 (1.140–24.459)	**0.033**	4.861 (1.042–22.667)	**0.044**
Primary tumor transverse diameter	1.776 (1.141–2.766)	**0.011**	2.273 (1.210–4.269)	**0.011**
Primary tumor AP diameter	1.837 (1.099–3.069)	**0.020**	1.274 (0.775–2.096)	0.339
Primary tumor ADC_mean_	28.548 (0.812–1003.839)	0.065	0.760 (0.002–293.529)	0.928
Primary tumor ADC_min_	25.267 (0.484–1320.121)	0.110	0.343 (0.001–112.207)	0.717
Muscle invasion (yes vs. no)	7.472 (1.612–34.624)	**0.010**	7.722 (0.862–69.145)	0.068
Invasion depth	2.025 (1.311–3.126)	**<** **0.001**	1.539 (0.965–2.453)	0.070
Metastasis volume	1.023 (0.982–1.065)	0.275	1.027 (0.984–1.072)	0.228
Metastasis volume (> 13 cm^3^ vs. ≤ 13 cm^3^)	1.398 (0.409–4.775)	0.593	1.390 (0.406–4.756)	0.600
Metastasis ENE	1.056 (0.309–3.608)	0.931	1.113 (0.322–3.843)	0.866
Metastasis ADC_mean_	6.631 (0.292–150.793)	0.235	3.924 (0.061–253.100)	0.520
Metastasis ADC_min_	54.659 (0.373–8003.590)	0.116	9.283 (0.018–4853.235)	0.485

*AP* anteroposterior,* ADC* apparent diffusion coefficient, *ENE* extranodal extension, *CI* confidence interval

Univariable analysis showed prognostic value for primary tumor volume, transverse diameter, anteroposterior diameter, muscle invasion, and invasion depth. After adjustment with p16, primary tumor volume had significant association with the LRR rate. This was a two-class variable, where tumors were divided by the median, to ≤ 7 cm^3^ and > 7 cm^3^. Primary tumor transverse diameter was also significantly associated with worse prognosis, whilst ADC values showed no significance. In p16-positive tumors, ADC values were significantly higher in grade 1–2 tumors (mean 0.883) compared to grade 3 tumors (0.736; *p* = 0.003). In these patients, tumor grade had no effect on treatment outcome.

### Differences in ADC Measurements Between Different MRI Systems

The ADC_myelum_ values in different MRI systems were significantly different between separate 1.5 T systems, and also between 1.5 T and 3 T systems (*p* = 0.007). After Bonferroni correction for multiple tests, the differences were no longer statistically significant.

### Interobserver Correlations

**Fig. 4:**
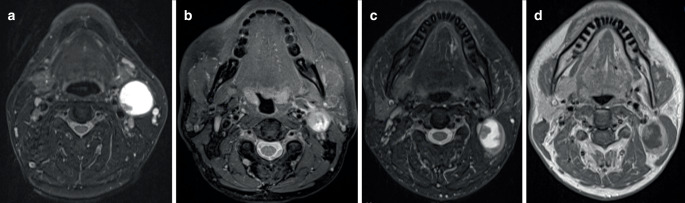
Examples of cystic and necrotic metastases. **a** T2 fat saturated image of a metastasis that was clearly cystic, with a thin capsule and homogeneous fluid, with only a minor wall irregularity. **b** Example of a necrotic node in a T2 fat saturated image, with a mainly irregular margin between fluid and solid parts. **c** The third example is of a metastasis that the radiologists graded differently. The margin appears smooth in T2 fat saturated image but (**d**) reveals more irregularity in T1 gadolinium-enhanced image, leading the other radiologist to grade the metastasis as necrotic. All three tumors were p16-positive

For the primary tumor the intraclass correlation coefficient (ICC) for ADC_mean_ was 0.783 and for ADC_min_ 0.797 and for metastasis 0.913 and 0.820, respectively. We observed a relatively inferior Cohen’s kappa (κ) interobserver agreement in evaluating necrotic and cystic nodes (κ = 0.499 and κ = 0.516, respectively, Table 2). Fig. 4 demonstrates examples of cystic and necrotic metastases.

## Discussion

To our knowledge this is the first study analyzing prognostic imaging factors in OPSCC and including a considerable number of p16 positive OPSCC patients. Our main finding was that our results do not support the hypothesis that ADC would be an independent prognostic factor. Higher ADC_mean_ and ADC_min_ were associated with lower DFS in univariable analysis but not after adjustment with the p16 status. This finding may be explained by the fact that ADC correlates with tumor p16 status [21, 22], which in itself is a strong predictor of disease recurrence and survival [31, 32].

Most studies evaluating the role of pretreatment ADC in predicting the results of (C)RT in different head and neck sites linked high ADC to worse prognosis [7, 9–16], while some showed no connection [17, 19, 33]. Interestingly, one study associated low ADC to worse 2‑year DFS, but over half of the patients were surgically treated [18]. Since previous studies have not differentiated results by site, it is hard to tell if ADC has real prognostic effect in cancers in other head and neck anatomic sites or if results are confounded because of the HPV-associated effects in ADC and survival among OPSCC.

In our study, among the p16 positive subgroup, ADC values were significantly higher in grade 2 tumors compared to grade 3 tumors. Similar observations have been previously reported in meningiomas [34], gliomas [35], and a nonsignificant correlation was found in oral and oropharyngeal cancers [36]. A study comparing histology to ADC values in laryngeal and hypopharyngeal cancers discovered that ADC correlated inversely with cell density, nuclear area, and nuclear-cytoplasmic ratio. ADC also correlated positively with the percentage area of stroma [37]. HPV-associated tumors are typically nonkeratinizing or only partially keratinizing, may have areas of central necrosis and cystic changes, and host tumor infiltrating lymphocytes. They often lack a strong stromal desmoplastic reaction and have a lower stromal volume [38]. Many of these characteristics can contribute to both lower ADC and better response to RT. HPV-associated cancer also has intrinsic genetic mechanisms, which might explain the radiation sensitivity [39]. Microscopic necrosis could also explain higher ADC, with hypoxia-initiating cellular survival mechanisms making the tumor less sensitive to RT, but so far hypoxia-related markers have not been found to be related to higher ADC [37, 40].

As presented in Table 2, even with a small number of patients, it appears that ADC values in p16-positive primary tumors and metastases were significantly lower compared to p16-negative tumors, which is in line with a previous systematic review and meta-analysis [22]. The p16-positive tumors were smaller and more often exophytic than not, similar to previous studies [41, 42]. Our cohort lacked any significant differences in the rates of cystic, necrotic, and solid metastases in relation to p16 status. This is in contrast to previous studies reporting a larger number of cystic metastases in p16-positive patients [26, 41, 42], while the limited number of p16-negative tumors might influence this assessment. Although cystic and necrotic nodes are clearly defined, in our study radiologists found the evaluation often challenging, which was reflected in the suboptimal interobserver correlation. On the contrary, other lymph node characteristics, such as ENE and matted nodes, showed better interobserver correlations.

We drew the ROI on the ADC maps with a free-hand method on the most central slice. We feel that this best resembles our available tools in everyday clinical practice and is easy to produce. The interobserver correlations for the ADC values were excellent. The ADC results in our study, however, may consequently differ from the studies that used automatic or semi-automatic segmentation. The different MRI systems used in this study bring forth variability in ADC values. These differences were observable in our measurements but were not statistically significant after Bonferroni correction for multiple tests.

We found a correlation between larger metastasis volume and worse DFS, regardless of p16 association. This is similar to a Finnish study comprising 91 OPSCC patients that found nodal volume to correlate with DFS and locoregional control both in p16-negative and p16-positive disease [8]. Davis et al. [43] also found that in HPV-positive OPSCC, smaller nodal volume led to better DFS. In our study, the LRR rate was higher in patients with a primary tumor volume over 7 cm^3^. The aforementioned reports failed to show similar results [8, 43]. In tonsillar tumors with unknown HPV association, the connection disappeared after taking the T‑stage into consideration [44]. Carpén et al. [8], however, observed a connection between larger primary tumor volume and worse OS in p16-negative disease. Interestingly, metastasis volume, but not primary tumor volume, correlated with survival in our study. This might be connected to our finding that larger neck metastasis volume but not larger primary tumor volume, correlated significantly with occurrence of later distant metastasis. A recent study showed that disease recurred only in OPSCC patients with persisting high risk HPV positivity after treatment [45]. In our hospital, we do not routinely determine the HPV status after treatment, although this might be worth considering in the future.

An unplanned break in radiation treatment led to worse DFS. The break duration was between 2–18 days, with most patients having mid-treatment breaks of under 10 days. In head and neck cancers, treatment breaks are known to decrease survival and local control [46, 47], tumor cell repopulation being faster during the break [48]. Incomplete radiotherapy seems to affect particularly the more radiosensitive HPV-associated oropharyngeal cancers [49].

In Finland surgery is usually the treatment of choice for patients with p16-negative tumors; however, (C)RT may be the option e.g., if surgery is expected to cause significant morbidity. We decided to include all patients treated with (C)RT with curative intent as our aim was neither a comparison of treatment modalities nor comparison of prognosis between p16-negative and p16-positive groups, and the final analysis accounted for the p16 status. This led to a small number of patients with p16-negative OPSCC in our cohort, and the recurrence rate and survival probably not being representative of the whole population with p16-negative tumors. Because of the small number of p16-negative OPSCC patients, and the generally good prognosis among the p16-positive OPSCC patients, event count remains fairly small and our survival analysis results must be interpreted with caution. To be able to obtain statistical power, we adjusted our survival analyses with p16 status only, as it is the most important factor affecting prognosis in OPSCC [31, 32]. One of the limitations in our study is use of ADC and p16 in the same Cox proportional hazards model as they have a strong statistical correlation; however, the observation that any prognostic impact of ADC remained absent in the p16-positive group supports our main finding of ADC being merely an indication of HPV status.

## Conclusion

Our study showed that in patients with OPSCC and with known p16 status, pretreatment tumor ADC values provide no additional benefit in evaluating prognosis after definitive (C)RT with curative intent. Our results indicate that ADC values should not be used to select patients for de-escalation trials or lighter treatment.
